# Autonomic Function in Fragile X Syndrome: A Systematic Review

**DOI:** 10.1111/jir.70106

**Published:** 2026-04-24

**Authors:** Sydni Weissgold, Sarah E. A. Eley, Damien Wright, Andrew G. McKechanie, Andrew C. Stanfield

**Affiliations:** ^1^ Patrick Wild Centre, Division of Psychiatry University of Edinburgh Edinburgh UK

**Keywords:** autonomic function, electrodermal activity, fragile X syndrome, heart rate variability, pupillometry

## Abstract

**Background:**

Fragile X syndrome (FXS) is a monogenic X‐linked cause of intellectual disability and autism. Individuals with FXS often have high levels of anxiety and sometimes display challenging behaviours. Autonomic dysfunction has been suggested to be one physiological mechanism that may contribute to these. Therefore, the objective of this review is to systematically examine existing evidence on autonomic function in FXS.

**Method:**

An electronic literature search was conducted on OVID platforms (Medline, Embase and PsycINFO) and Web of Knowledge databases for references published before 22 April 2025, which physiologically measured autonomic function in FXS. A preregistered search strategy was designed to gather literature on the autonomic nervous system in FXS.

**Results:**

A total of 1426 articles were identified, and 28 studies met the inclusion criteria. Samples comprised individuals across the lifespan (ages < 1–71 years), with most studies utilising a case control design to examine indices of autonomic function; 75% of studies found a between‐group difference in autonomic function metrics. Of these studies, hyperarousal in the FXS group was present in 86% (*N* = 18) of studies. Although some studies reported associations of autonomic function with clinical characteristics, findings varied considerably between studies. There was some evidence of potential differences between genders, although more research in female populations is required.

Of the 28 included studies, 64% (*N* = 18) further examined links between autonomic function and clinical characteristics associated with FXS, demonstrating links between relevant clinical symptoms, that is, autistic traits and hyperarousal, as well as potential gender differences in autonomic function.

**Conclusions:**

The findings demonstrate evidence for autonomic hyperarousal as a clinical phenotype in FXS across the lifespan. Future work is required to define whether overactivation of the sympathetic nervous system, as indicated by hyperarousal in FXS, can be linked to clinical symptoms. Variations in sample demographics as well as in methodological approaches to measuring autonomic function both hinder accurate comparison of the results from included studies.

## 1 Background

Fragile X syndrome (FXS) is a leading genetic cause of intellectual disability (ID), with an estimated prevalence of up to 1.49 in 10 000 males and 0.82 in 10 000 females (Fisher et al. 2025). A trinucleotide (CGG) repeat expansion on the fragile X messenger ribonucleoprotein 1 (FMR1) gene, which is located on the long arm of the X chromosome, is responsible for FXS syndrome (Hagerman et al. 2017). This trinucleotide repeat leads to methylation of the *FMR1* promoter region and subsequent reduced production of fragile X messenger ribonucleoprotein (FMRP).

Along with ID (approximately 80% prevalence in FXS) and cognitive deficits, the FXS phenotype is associated with autism, sensory sensitivities, mild motor delays and language delays (Maes et al. 2000; Garber et al. 2008). Anxiety, hyperactivity and challenging behaviours, such as aggression, are often present in individuals with FXS as well (Hagerman and Hagerman 2002; Garber et al. 2008; Tsiouris and Brown 2004; Hardiman and Mcgill 2018). Reports suggest that aggressive behaviours, such as hand‐biting, arise from sensory overstimulation and subsequent behavioural hyperarousal (Hardiman and Mcgill 2018; Miller et al. 1999). The phenotype in FXS is highly variable, due in part to differences in FMRP production (Loesch et al. 2004; Tassone et al. 1999), with females in particular being less severely affected than males, as a result of random X inactivation. Variation in methylation of the *FMR1* promoter region is also associated with differences in presentation due to the effect on FMRP levels, with those individuals who show methylation mosaicism being less severely affected than those without (Meng et al. 2022).

Emerging evidence suggests that autonomic dysfunction may be an additional characteristic associated with FXS. The autonomic nervous system (ANS) is a branch of the peripheral nervous system, which regulates homeostasis and physiological responses to the internal and external environment via sympathetic and parasympathetic branches. Higher sympathetic activity is present when an individual is experiencing stress, as opposed to increased vagal tone, that is, parasympathetic activity, when an individual requires rest. Autonomic function can be measured via examination of downstream physiological indices of sympathetic or parasympathetic activation, such as cardiac, electrodermal or pupillary arousal. Such metrics can indicate whether an individual is experiencing increased sympathetic input and/or decreased parasympathetic input, both of which could indicate autonomic hyperarousal.

The first empirical investigation of the ANS in FXS was published by Belser and Sudhalter (1995), who demonstrated autonomic hyperarousal via increased skin conductance levels (SCLs) in males (ages 6–16 years) with FXS. Since this time, there have been multiple articles published, using a variety of methods in varying populations, including both males and females, and covering a wide age range from infancy to adulthood (Keysor et al. 2002; Hogan et al. 2021; Miller et al. 1999; Hall et al. 2012). Links between hyperarousal of the ANS and emotions, stress and behaviour in other genetic‐based ID's have also been described. For example, emotional, behavioural and autonomic dysregulation (EBAD), a presentation described in Rett syndrome, may help to explain associations between unrestrained vagal tone with emotional and behavioural problems (Singh and Santosh 2018). It is possible, therefore, that autonomic dysfunction, and in turn, the absence of physiological homeostasis, may contribute to or underlie behavioural characteristics present in FXS.

We therefore set out to systematically synthesise existing evidence regarding autonomic function in FXS. In doing so, we aimed to determine first whether there is altered autonomic function in FXS and, if so, whether sympathetic, parasympathetic or both processes are affected. We also aimed to identify whether autonomic function is associated with clinical symptoms observed in FXS.

## 2 Methods

### 2.1 Search Strategy

We conducted an electronic search on OVID platforms (Medline, Embase and PsycINFO) and Web of Science databases for any article published between 1991 and 22 April 2025 (date of the search); 1991 was selected as the start date as this is when modern genetic testing for FXS began (Macpherson and Murray 2016).

Search terms included keywords, using MeSH terminology if applicable, in the fields of autonomic function, for example, ‘vagal tone’, ‘sympathetic nervous system’ or ‘heart rate variability’. In addition, we included keywords related to FXS, such as ‘fragile X syndrome’, ‘FXS’ and ‘intellectual disabilit*’. The complete search strategy can be seen in the Supporting Information (A). The protocol for this systematic review was pre‐registered on Prospero (CRD420251035098) to ensure transparency and minimise bias. The protocol was amended once to include a search of the references in studies that meet the inclusion criteria.

### 2.2 Inclusion and Exclusion Criteria

Articles were included if they measured autonomic function (via cardiac measures, electrodermal activity and pupillary function) and included human participants of all ages with a diagnosis of FXS. If any of the following criteria were met, the reference was excluded: (1) animal studies, (2) autonomic function only assessed via behavioural measures, for example, self‐ or informant‐report questionnaires; (3) individuals who did not have a diagnosis of FXS or had the FXS premutation; (4) non‐availability of English version of the reference; or (5) reviews (systematic, narrative or meta‐analyses), conference abstracts or dissertations.

### 2.3 Study Screening and Extraction

References identified in the search were uploaded into Covidence, an electronic software platform developed to facilitate the production of systematic literature reviews (Covidence 2024). Duplicate articles were removed by Covidence initially, and further duplicates were removed manually. Titles and abstracts of all references were screened by two reviewers (S.W. and S.E.) against the inclusion criteria. Next, two reviewers (S.W. and S.E.) reviewed the full text of all remaining articles. Disagreements between reviewers at the title and abstract, as well as full‐text, screening stages were resolved through discussion; if an agreement could not be reached, the decision was made by a third reviewer (A.S.).

Data from references which met the inclusion and exclusion criteria were extracted on Covidence by two reviewers (S.W. and S.E.). The following study information was extracted into an excel spreadsheet: authors, year of publication, condition studied, place of publication, diagnostic methods, objective/aims of the study, hypotheses, study type (e.g., case control and longitudinal), comparison group used, sample size, age range, gender, exclusion criteria, autonomic domain measured, study protocol, recording instrument, recording time, analytic metric used and key findings. Missing information was noted as N/A.

### 2.4 Quality Assessment

To critically appraise the quality of the references included in this review, we used the Newcastle–Ottawa scale (Wells et al. 2013) for its structured assessment of case control studies. The quality of the study design was assessed by two reviewers (S.W. and S.E.). Studies were examined on whether they identified and/or accounted for necessary confounding variables; confounders to be included in these articles are age, gender and medication use. Disagreements between reviewers at the title and abstract, as well as full‐text, screening stages were resolved through discussion; if an agreement could not be reached, the decision was made by a third reviewer (A.S.).

## 3 Results

**FIGURE 1:**
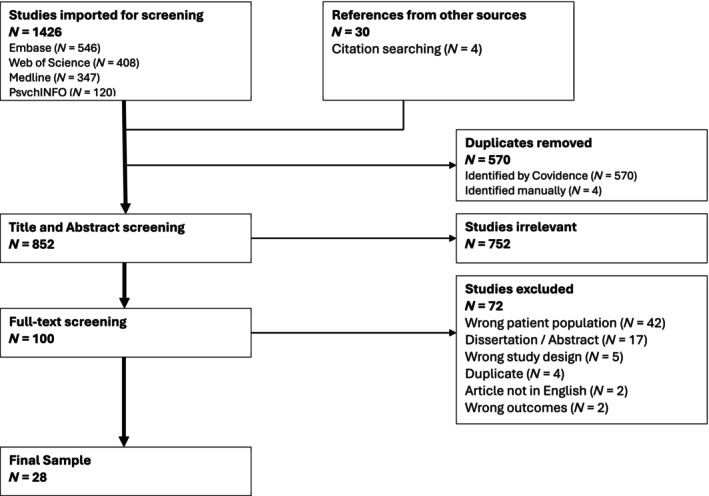
Flowchart detailing the number of studies screened, included and excluded in this systematic review.

The initial search identified 1426 studies from Ovid platforms (Medline [*N* = 347], Embase [*N* = 546], PsycINFO [*N* = 120] and Web of Science [*N* = 408]; Figure 1). After removal of duplicates (*N* = 570 identified by Covidence, *N* = 4 identified manually), title and abstract screening (*N* = 850) and full‐text screening (*N* = 98), 26 articles met the inclusion criteria. The reference lists of the 26 included articles were screened; two additional studies met the inclusion criteria, bringing the total number of included studies to 28 (comprising a total of 727 participants). For a description of physiological metrics used to measure autonomic function, see Arora et al. (2021).

### 3.1 Study Characteristics: Sample, Procedure and Findings

Of the 28 identified articles examining autonomic function in FXS, 68% (*N* = 19) utilised cardiac measures, 25% (*N* = 7) recorded electrodermal activity (EDA), and 18% (*N* = 5) measured pupil dilation (Table 1). Of these articles, 11% (*N* = 3) examined autonomic function using a multimodal approach, that is, cardiac and EDA assessments. The recording time across 25 studies ranged from 1.5 to 40 min; data were not available from the remaining three articles.

**TABLE 1:** Fragile X syndrome (FXS): Included study characteristics.

First author (year)	Diagnostic methods	Study design	Sample characteristics				ANS measure	Metrics reported
			*FXS: N (M:F)*	*Comparison group condition*	*Comparison group: N (M:F)*	*Age range*		
Baranek et al. 2008	Genetic report	Cohort Study	13 (13:0)	N/A	N/A	0.75–4.5 years (9–54 months)	Cardiac	IBI, vagal tone
Belser and Sudhalter 1995	Not stated	Case control	2 (2:0)	ADHD & Down syndrome	2 (2:0)	Range = 6–16 years	EDA	SLC (uS)
Black et al. 2021	Genetic report	Case control	32 (22:10)	Low‐risk controls	41 (32:9)	1 year (12 months)	Cardiac	RSA, RSA reactivity
Boccia and Roberts 2000	Not stated	Case control	20 (20:0)	TDC	20 (20:0)	Mean = 4 years	Cardiac	IBI, natural logarithm of frequency values to get vagal and sympathetic tone
Cohen et al. 2015	Genetic report	Case control	FXS: 12 (12:0) FXS + ASD: 17 (17:0)	ASD & TDC	ASD: 12 (12:0) TDC: 11 (11:0)	10–17 years	EDA & cardiac	EDA (frequency, amplitude, speed of response), HRV (IBI, frequency values)
Ezell et al. 2021	Genetic report	Case control	FXS: 21 (11:10) FXS + ASD: 17 (13:4)	ASD & TDC	ASD: 42 (36:6) TDC: 27 (20:7)	3–6 years (36–72 months)	Cardiac	RSA, IBI
Farzin et al. 2009	Not specified	Case control	16 (13:3)	TDC	16 (13:3)	10–23 years	Pupil	Mean pupil diameter, pupil reactivity
Farzin et al. 2011	Genetic report	Case control	15 (12:3)	TDC	20 (10:10)	7–71 years	Pupil	Mean pupil diameter, pupil reactivity
Hagerman et al. 2002	Genetic report	Case control	19 (13:3)	ADHD/DD	17 (12:5)	5–16 years	EDA	Mean number of EDR peaks, log amplitude of main peak, EDR frequency
Hall et al. 2009	Genetic report	Case control	50 (26:24)	TDC siblings	50 (26:24)	7–23 years	Cardiac	IBI, vagal tone, HR variance
Hall et al. 2012	Genetic report	RCT	8 (8:0)	N/A	N/A	13–28 years	Cardiac	HR, HRV, RSA
Heilman et al. 2011	Genetic report	Case control	12 (12:0)	TDC	17 (17:0)	6–23 years	Cardiac	HR, RSA, respiration rate
Hessl et al. 2019	Genetic report	RCT	57 (90% Male)	N/A	N/A	12–45 years	Pupil	Mean pupil diameter, pupillary response
Hogan et al. 2021	Genetic report	Case control	73 (73:0)	TDC	79 (79:0)	0.25–6.9 years (3–83 months)	Cardiac	IBI, RSA
Keysor et al. 2002	Genetic report	Case control	13 (0:13)	Turner TDC	Turner: 11 (0:11) TDC: 14 (0:14)	12–22 years	EDA & cardiac	Mean and range of SCL, SCL fluctuations, IBI, RSA
Klusek et al. 2013	Unclear	Case control	39 (39:0)	ASD TDC	ASD: 40 (40:0) TDC: 27 (27:0)	4–15 years	Cardiac	IBI, RSA
Li et al. 2022	Unclear	Case control	35 (0:35)	TDC	30 (0:30)	Mean FXS age: 11 years Mean TDC age: 10 years	Pupil	Pupil diameter
Miller et al. 1999	Genetic report	Case control	A: 25 (19:6) B: 15 (unclear)	TDC	A: 25 (matched) B: 15 (unclear)	4–49 years	EDA	EDR magnitude, mean number of peaks
Miller et al. 2024	Genetic report	Case control	32 (0:32)	Non‐FXS (ID/DD)	23 (0:23)	6–16 years	Pupil	Mean pupil diameter
Roberts et al. 2001	Unclear	Case control	27 (27:0)	TDC	31 (31:0)	1–11 years	Cardiac	IBI, LF, HF, LF/HF
Roberts et al. 2006	Unclear	Case control	29 (29:0)	TDC	31 (31:0)	1–11 years	Cardiac	LF, logHF, vagal tone
Roberts et al. 2008	Unclear	Case control	13 (0:13)	Turner TDC	Turner: 11 (0:11) TDC: 14 (0:14)	12–22 years	EDA & cardiac	IBI, RSA amplitude, SCL
Roberts, Hatton, et al. 2012	Genetic report	Case control (longitudinal)	13 (13:0)	TDC	12 (12:0)	9, 12 & 18 months (TDC = 12 months only)	Cardiac	Mean HR, HR SD, decelerative and accelerative HR components
Roberts, Tonnsen, et al. 2012	Genetic report	Case control (longitudinal)	31 (31:0)	TDC	25 (25:0)	0.6–3.3 years (8–40 months)	Cardiac	IBI, vagal tone
Roberts et al. 2013	Genetic report	Case control (cross‐sectional)	22 (22:0)	TDC	27 (27:0)	1–10 years	Cardiac	Mean IBI, IBI reactivity
Tonnsen et al. 2013	Genetic report	Case control	21 (21:0)	TDC	19 (19:0)	1–4.8 years (12–58 months)	Cardiac	IBI, RSA
Wall and Roberts 2023	Genetic report	Case control	41 (29:12)	ASD TDC	ASD: 47 (40:7) TDC: 32 (25:7)	3‐5 years	Cardiac	RSA
Williams et al. 2013	Genetic report	Case control	12 (0:12)	CA MA	CA: 12 (0:12) MA: 12 (0:12)	5–38 years	EDA	Mean SCR magnitude and frequency

Abbreviations: ADHD (attention‐deficit/hyperactivity disorder), ASD (autism spectrum disorder), CA (chronological age), DD (developmental delay), EDA (electrodermal activity), EDR (electrodermal response), FXS (fragile X syndrome), HF (high frequency), HR (heart rate), HRV (heart rate variability), IBI (interbeat interval), ID (intellectual disability), LF (low frequency), LF/HF (high–low frequency ratio), logHF (natural logarithm of high frequency), MA (mental age), RCT (randomised control trial), RSA (respiratory sinus arrhythmia), SCL (skin conductance level), TDC (typically developing controls), SD (standard deviation).

### 3.2 Study Populations

The age ranges across the studies ranged from < 1 year (3 months) to 71 years of age, with two studies examining autonomic function in infancy (ages 0–2 years), nine during childhood (ages 3–18 years) and none solely including adults (18 + years). However, seven articles included a combined sample of participants in infancy and childhood and *N* = 10 included children and adults. Consistent with the increased severity of the FXS clinical profile in males compared to females (Klusek et al. 2014), 14 studies included males only, five studies solely included females, and nine included both males and females. The sample size of the FXS group across studies ranged from 2 to 73 individuals, with 25 utilising a case control study design; two studies were randomised control trials (RCTs), one study was a cohort study, and two of the case control studies used longitudinal methods as well. Of the studies which recruited a comparison group, typically developing controls (TDC) were used in 22 of the studies, including chronological and mental age–matched controls (*N* = 1). Several of these studies recruited an autistic cohort (*N* = 4) or a cohort with Turner syndrome (*N* = 2) in addition to the TDC group. Three case control studies solely included a comparison group, which were not typically developing attention‐deficit/hyperactivity disorder (ADHD) (*N* = 2), idiopathic ID and/or developmental delay (DD) (*N* = 2) and Down syndrome (*N* = 1), sometimes including more than one comparison population within a single study. These data can be viewed in Table 1.

### 3.3 Study Procedures

Details relating to study protocol and key findings for each study which examined autonomic function in FXS can be viewed in Table 2. Three studies measured autonomic function solely at rest (e.g., sitting quietly or passively watching a film), whereas the majority of studies (*N* = 25) utilised, often in conjunction with a baseline condition, an experimental paradigm that included social tasks (i.e., conversations with a researcher in which eye contact was manipulated) (Klusek et al. 2013), viewing faces of varying emotional content (Farzin et al. 2011), sensory stimuli presentation (visual, auditory and olfactory included) (Hagerman et al. 2002), unstructured toy play or cognitive tasks (Roberts et al. 2008).

**TABLE 2:** Protocol and key findings from included studies.

First author (year)	ANS measure	Study paradigm	Key finding	Summary of findings
Baranek et al. 2008	Cardiac	Baseline (quiet play with toys)	No between‐group differences in HRV	Neither IBI nor vagal tone was predictive of hyperresponsiveness or hyporesponsiveness to sensory stimuli.
Belser and Sudhalter 1995	EDA	Conversations with and without eye contact	Increased sympathetic activation in FXS	Higher SCL levels in the FXS group were seen during eye contact condition; this effect was not observed in the comparison group.
Black et al. 2021	Cardiac	Baseline and stranger approach task	Lower HRV in FXS	FXS infants did not show the reduction in RSA between tasks that was seen in TDC, i.e., a blunted RSA response in FXS.
Boccia and Roberts 2000	Cardiac	Passive (watching a video) and active (cognitive) tasks	Decreased vagal tone in FXS	Boys with FXS had shorter IBI, lower vagal tone and different patterns of arousal compared to TDC.
Cohen et al. 2015	EDA & cardiac	Viewing emotional images paired with an auditory stimulus	Increased sympathetic activation in FXS	Hyperresponsive sympathetic activity in FXS & FXS + ASD groups (not seen in ASD or TDC groups); ASD & FXS + ASD groups showed decreased parasympathetic activity compared to FXS & TDC groups.
Ezell et al. 2021	Cardiac	Auditory stimuli presented	No autonomic response from the FXS group	Neither the FXS group showed a difference in cardiac response pre and poststimulus. A positive relationship was observed between nonverbal mental age and autistic traits with IBI in the FXS group; the relationship between parent‐reported anxiety and cardiac measures was non‐significant.
Farzin et al. 2009	Pupil	Images of scrambled and assembled faces shown	Increased sympathetic activation in FXS	Increased pupillary reactivity was seen in FXS compared to TDC when viewing emotional faces. Pupil size did not differ by sex in FXS, nor was pupil size associated with autistic traits.
Farzin et al. 2011	Pupil	Images of scrambled and assembled faces shown	Increased sympathetic activation in FXS	FXS demonstrated greater pupillary dilation in response to faces relative to controls.
Hagerman et al. 2002	EDA	Sensory stimuli presented	Increased sympathetic activation in FXS	No group differences in baseline EDR; FXS demonstrated a decrease in EDR amplitude and frequency when treated with stimulants compared to TDC.
Hall et al. 2009	Cardiac	Baseline and conversation with eye contact	Decreased HRV and vagal tone in FXS	Lower heart rate, baseline vagal tone and HRV in FXS compared to TDC. Baseline vagal tone and HRV were lower in females with FXS compared to TDC females; higher FMRP levels were associated with increased HRV in FXS females. Neither eye gaze avoidance nor medication usage was associated with cardiac measures.
Hall et al. 2012	Cardiac	Structured social challenge	No ANS response to medication	No differences found between medication and placebo.
Heilman et al. 2011	Cardiac	Social challenge, stranger introduction & auditory stimuli	Increased sympathetic activation in FXS	Compared to TDC, the FXS group had faster baseline HR, a decrease in RSA with age and showed an atypical increase in RSA during social challenge.
Hessl et al. 2019	Pupil	Viewing scrambled and assembled faces	Pupil response to medication	Greater pupil reactivity after treatment with Mavoglurant.
Hogan et al. 2021	Cardiac	Watched an animated film	Increased sympathetic activation in FXS	Physiological hyperarousal present by 24 months; atypical ANS development in FXS compared to TDC. Reduced RSA predicted autistic traits, but not anxiety symptoms, in FXS and TDC groups at 24 months. Shorter IBI at 24 months predicted anxiety symptoms in FXS and TDC males later in childhood.
Keysor et al. 2002	EDA & cardiac	Baseline and cognitive tasks	Increased sympathetic activation in FXS	FXS displayed higher SCL during baseline than the Turner/TDC groups; FXS exhibited increased arousal during the divided attention task.
Klusek et al. 2013	Cardiac	Baseline and conversation with the researcher	Increased sympathetic activation in FXS	FXS had lower IBI than the TDC group but not than the ASD group; the FXS + ASD group had lower IBI compared to the FXS‐only group. Cardiac measures were not associated with autism severity, nor anxiety symptoms. A positive relationship was observed between the pragmatic language task and vagal tone in males with FXS.
Li et al. 2022	Pupil	Structured conversation task with and without eye contact	Pupil response to task observed	FXS has a significantly larger pupil diameter during eye contact compared to the non‐eye contact condition; group differences are not stated.
Miller et al. 1999	EDA	Sensory stimuli	Increased sympathetic activation in FXS	The FXS group had higher EDR magnitude, more EDR responses per stimulation and lower habituation rate than the TDC group. EDR magnitude and number of responses are negatively associated with FMRP levels.
Miller et al. 2024	Pupil	Conversations with and without eye contact	Increased sympathetic activation in FXS	Compared with the non‐FXS group, FXS showed a higher mean pupil diameter during eye contact condition.
Roberts et al. 2001	Cardiac	Passive (watching TV and active [cognitive] tasks)	Increased sympathetic activation in FXS	FXS had higher sympathetic activity; compared to the TDC group, FXS did not show adaptations in IBI length or vagal tone based on phase, whereas TDC did adapt. FMRP levels were not associated with cardiac activity.
Roberts et al. 2006	Cardiac	Baseline and cognitive tasks	Decreased vagal tone in FXS	FXS had lower baseline vagal tone and higher vagal suppression than TDC. Group differences in temperament were mediated by vagal tone.
Roberts et al. 2008	EDA & cardiac	Baseline and cognitive tasks	Hypoarousal via SCL in FXS	No differences between groups on cardiac measures; FXS showed a lower change in SCL between conditions compared to the Turner/TDC groups. Electrodermal activity, but not cardiac activity, was associated with poor performance on mental arithmetic tasks in the FXS group.
Roberts, Hatton, et al. 2012	Cardiac	Unstructured play with a toy	Lower HRV in FXS	FXS had lower HRV and shallower decelerations compared to TDC infants at 12 months. Neither the Mullen composite score nor autistic traits were associated with heart rate.
Roberts, Tonnsen, et al. 2012	Cardiac	Toy play and arm restraint procedure	Decreased vagal tone in FXS	Compared to TDC, FXS showed lower vagal tone, shorter IBI and less IBI modulation during toy play. A significantly negative relationship between vagal tone and autistic traits was present from 22 months of age.
Roberts et al. 2013	Cardiac	Baseline (watching a video) and auditory stimulus	Increased sympathetic activation in FXS	Boys with FXS display increased cardiac reactivity across ages compared to TDC. A significant relationship was observed between mental age, but not behaviour, with cardiac reactivity in FXS.
Tonnsen et al. 2013	Cardiac	Stranger approach paradigm	Age‐dependent changes in IBI in FXS	FXS demonstrated age‐dependent changes in IBI across phases; no group‐by‐phase interactions. Distress vocalisations were predicted by IBI and RSA in FXS.
Wall and Roberts 2023)	Cardiac	Baseline (video without words)	No between‐group differences in RSA	Baseline RSA did not differ across groups. Baseline RSA and autism severity were significantly negatively correlated in the FXS group but not in the TDC group.
Williams et al. 2013)	EDA	Emotional visual stimuli	Increased sympathetic activation in FXS	The FXS group had higher SCR magnitude during socially salient stimuli compared to CA; there were no differences between groups for SCR frequency.

Abbreviations: ASD (autism spectrum disorder), CA (chronological age–matched controls), EDA (electrodermal activity), EDR (electrodermal response), FMRP (fragile X messenger ribonucleoprotein 1 gene), FXS (fragile X syndrome), HRV (heart rate variability), IBI (interbeat interval), RSA (respiratory sinus arrhythmia), SCL (skin conductance level), SCR (skin conductance response), TDC (typically developing controls).

### 3.4 Findings

Seventy‐five per cent (*N* = 21) of studies (comprising 514 of 727 participants) demonstrated differences between FXS participants and the comparison group. The samples in these studies ranged in age from 3 months to 71 years and included solely males (*N* = 11), solely females (*N* = 4) and a combination of male and female participants (*N* = 6). The comparison groups included in these studies were TDC (*N* = 13), a combination of TDC and ASD groups (*N* = 3), a combination of TDC and Turner syndrome groups (*N* = 2), ID/DD (*N* = 1) and ADHD/Down syndrome (*N* = 2) samples.

Of the remaining 25% (*N* = 7) of studies, which did not demonstrate between‐group differences, four were case control studies that did not find statistically significant differences between groups (135 participants, age range: 1–11 years; comparison groups included: TDC [*N* = 2] and a combined sample of TDC and ASD groups [*N* = 2]). One of these studies demonstrated that autonomic function in FXS is associated with age, specifically demonstrating a significant relationship between chronological age and cardiac indices of autonomic function (Tonnsen et al. 2013) but did not show between‐group differences. Lastly, three of the studies that did not report significant findings related to autonomic function between groups were RCTs (*N* = 2) or cohort studies (*N* = 1) and therefore did not include a comparison group (Hall et al. 2012; Baranek et al. 2008; Hessl et al. 2019). One RCT determined that intranasal oxytocin did not influence cardiac measures of autonomic function in children and adults with FXS (Hall et al. 2012), and the other demonstrated increased pupil diameter in children and adults with FXS during a course of mavoglurant treatment (Hessl et al. 2019). The cohort study demonstrated that neither IBI nor vagal tone was predictive of hyperresponsiveness or hyporesponsiveness to sensory stimuli (Baranek et al. 2008).

Of the 21 studies which reported a between‐group difference in autonomic function, 18 demonstrated autonomic hyperarousal in the FXS sample (comprising 465 participants), whereas only one study demonstrated hypoarousal (13 participants) and two studies reported decreased heart rate variability (HRV) (a total of 29 participants). Autonomic hyperarousal was primarily indicated by increased sympathetic tone, although there were four studies which reported either decreased vagal tone or increased autonomic reactivity. Three of these four studies were conducted in exclusively paediatric populations (ages of 0.6–11 years), whereas the remaining study included children and young adults (ages 7–23 years). Autonomic hypoarousal, indicated by decreased SCL, was found in one study (*N* = 13 participants) using cardiac and EDA measures. Decreased HRV was found as well in two studies, both using cardiac measures.

Several studies (*N* = 18; 64%) examined the link between autonomic function and clinical characteristics of FXS, such as sex, age, autistic traits, anxiety and cognitive abilities. Of the studies that examined these links, it was common for autonomic function to be examined alongside several outcome measures.

Of those which statistically investigated the association of age and gender with autonomic function, the results varied between studies. Statistically significant relationships between autonomic function with age and sex were reported in some studies (*N* = 2 and *N* = 1, respectively) but not in others (*N* = 2 and *N* = 2, respectively), indicating a lack of consensus around this issue. Notably, however, the only study to find hypoarousal was conducted in a cohort of 13 females with FXS (Roberts et al. 2008). Also, three of the four studies that demonstrated hyperarousal via decreased parasympathetic activity and/or decreased vagal tone, recruited only male participants; the fourth study recruited both male and female participants (Boccia and Roberts 2000; Hall et al. 2009; Roberts et al. 2006; Roberts, Tonnsen, et al. 2012).

In terms of clinical features, significant relationships were seen between autonomic function and autistic traits (*N* = 4), anxiety (*N* = 1), mental age (*N* = 2), cognition (*N* = 2), sex (*N* = 1), medication use (*N* = 1), outcomes on social tasks (*N* = 2) and temperament (*N* = 1). Largely, these reports demonstrate that the relevant clinical characteristics (i.e., autistic traits and anxiety) were associated with hyperarousal of the ANS in FXS. Other studies, however, did not identify such relationships, again indicating lack of consensus: autistic traits (*N* = 4), anxiety (*N* = 3), sex (*N* = 2), age/developmental age (*N* = 2), social task outcomes (*N* = 2), cognition (*N* = 2), sensory processing (*N* = 1) and medication usage (*N* = 1).

Three studies reported whether the measure of autonomic function was associated with FMRP levels. Two found a positive relationship between autonomic metrics (cardiac and electrodermal) and FMRP levels; the third found that FMRP was not significantly associated with heart activity.

### 3.5 Quality Assessment

The quality assessment of individual studies can be viewed in the Supporting Information (B). Broadly, across the studies identified in this review, the quality analysis identified that most studies used appropriate statistical analysis strategies and outcome measures. Matching of case control groups was one potential issue identified by the quality analysis. All case control studies (*N* = 25) matched for at least age, gender or medication; however, only nine studies matched for all three. Thirteen studies matched for at least two of the necessary confounders (most commonly age and gender, without controlling for medication use), and only three accounted for one confounding variable. The studies that were not case control in design (e.g., RCT/cohort study; *N* = 3) were not assessed using the Newcastle–Ottawa Scale (Baranek et al. 2008; Hall et al. 2012; Hessl et al. 2019). However, all three were deemed to be of good quality, demonstrated by the use of independent genetic testing to confirm FXS diagnosis and limited evidence of bias (however, sampling bias may be present in one study). Furthermore, if nonrespondents were present in the study, they were described.

## 4 Discussion

This study presents the first pre‐registered systematic review of autonomic function in FXS, demonstrating that autonomic hyperarousal is a core phenotypic feature. A systematic search identified 28 articles that examine autonomic function in FXS using cardiac, pupillary and electrodermal modalities. Eighteen of 28 studies covering 465 people found autonomic hyperarousal, indicated via increased sympathetic or decreased parasympathetic input. Only one study covering 13 people reported hypoarousal, whereas four case control studies that examined between‐group differences (135 participants) reported no differences in autonomic function between groups. Therefore, on balance, the predominant finding is one of autonomic hyperarousal. Studies that found between‐group differences included samples across the lifespan, suggesting that autonomic dysfunction is not limited to any specific group of people with FXS.

The wide age ranges recruited by the included studies (3 months – 71 years of age) that found between‐group differences indicate that altered autonomic function in FXS may be present from as early as infancy and may persist through to adulthood. There was some evidence that the studies which observed decreased parasympathetic activity and/or a reduction in vagal tone, as opposed to increased sympathetic input, were more likely to have been conducted in a younger sample (i.e., infants, toddlers and children). However, increased sympathetic activity was also observed in samples including infants through to older adults. Notably, the majority of research studies included in this review recruited either solely children or were mixed groups of children and adults, with none of the included studies focusing solely on autonomic function in adults with FXS. Given that paediatric FXS populations, particularly those with comorbid autism diagnoses, are reported to display increased levels of challenging behaviours and sleep problems, age‐sensitive investigations of autonomic function in FXS are warranted (Verdura et al. 2021). Furthermore, four studies included in this review investigated the relationship between the autonomic metrics collected with age; two report a statistically significant relationship, and two report nonstatistically significant findings. Given that autonomic function varies by age in typically developing populations (Parashar et al. 2016), it is critical to prospectively examine the relationship between autonomic function and age within childhood, adolescence and adulthood in FXS.

50% of FXS studies solely examined males; this is expected as FXS is an X‐linked disorder and therefore commonly more prevalent and severe in males than in females (Klusek et al. 2014). The remaining 50% examined either solely females or a mixture of both genders. Hyperarousal via decreased parasympathetic activation and/or a reduction in vagal tone was mostly observed in studies which included exclusively male participants (3 of 4 studies). Given that males are typically more profoundly affected by FXS than females, it is possible that reduced parasympathetic activity is associated with the increased severity of FXS symptoms seen in this group. A direct examination of how autonomic function is affected by gender in FXS would be helpful to clarify whether females with FXS also show parasympathetic dysfunction, especially in those who are more severely affected by FXS. However, it is important to note that significant heterogeneity exists within males with FXS, including the level of cognitive impairment (Verdura et al. 2021). Whether autonomic hyperarousal is associated with the level of impairment and severity of the condition, or gender, is an important distinction for future studies to investigate. Three studies in this review examined the relationship between sex and autonomic outcome measures (two found non‐significant results and one reported a significant association); however, the small sample and contradictory findings result in a lack of clarity of the intricacies of autonomic function in FXS in both genders.

Several of the studies that solely recruited females with FXS did report differences in autonomic function between cases and controls, indicating that dysregulated autonomic function may not be sex specific. Of the case control studies that solely included female participants and found between‐group differences, 75% demonstrated autonomic hyperarousal via increased sympathetic activation. However, the only study in this systematic review to demonstrate autonomic hypoarousal in FXS was conducted with solely female participants, further demonstrating the need for targeted investigations of the effects of gender on autonomic function in FXS.

Only three studies specifically examined the relationship between FMRP levels and autonomic measures (Hall et al. 2009; Miller et al. 1999; Roberts et al. 2001). These showed varying relationships, with one study demonstrating increased FMRP levels associated with increased HRV, another demonstrating a negative relationship between EDR magnitude and FMRP levels, and the third did not find a statistically significant relationship between cardiac activity and FMRP levels. It is important that future studies quantify FMRP and related measures such as *FMR1* methylation mosaicism, as these are known to be associated with phenotypic variability in FXS (Meng et al. 2022). It is possible that the heterogeneity between studies in the current review may relate to differences in FMRP levels between studies.

Ninety per cent of the between‐group differences found in case control studies demonstrated a pattern of autonomic hyperarousal, that is, underactivation of the parasympathetic nervous system and/or overactivation of the sympathetic nervous system. Only 5% of studies found the opposite pattern, hypoarousal, in FXS. This highlights that autonomic hyperarousal is likely to be the predominant presentation in FXS; however, this is by no means universal. Autonomic hyperarousal may be linked with the clinical phenotype of FXS, as has been suggested for other neurodevelopmental conditions, most notably in Rett syndrome. A link between EBAD in Rett syndrome, a progressive neurodevelopmental condition caused by de novo mutations on the MECP2 gene, has been described (Weaving et al. 2005). Autonomic dysfunction is present in 75% of Rett syndrome patients and is associated with physical symptoms, including abnormal breathing patterns and arrhythmias (Gualniera et al. 2021). Further emotional dysregulation may be linked to autonomic dysfunction in Rett syndrome, which can be demonstrated via anxiety, severe panic attacks and/or sleep disturbances (Gualniera et al. 2021).

Findings from the studies included in this systematic review suggest that a similar relationship between emotions, behaviour and autonomic hyperarousal may be present in FXS. However, although not discussed in all included studies, those studies that did examine relationships between clinical symptoms and autonomic hyperarousal were somewhat contradictory. Many studies reported statistically significant relationships between autonomic function and clinical outcomes including autistic traits and anxiety, while others did not find statistically significant relationships. Although some of this inconsistency may relate to statistical power, variations in assessment methodology for both autonomic function and clinical outcomes may also contribute to these contradictory results. Given the close links between anxiety and autonomic dysfunction (Hu et al. 2016), autism and autonomic dysfunction (Kushki et al. 2014), as well as the high prevalence rates of anxiety in FXS (Cordeiro et al. 2011), further targeted research in large populations is warranted to elucidate this link.

The methods and demographics of included studies varied widely. For instance, the sample size of included studies ranged from 2 to 73. The studies which included fewer participants, for example, *N* = 2, are unlikely to be sufficiently statistically powered, and the results should be considered with caution. Furthermore, there was variation in the selected comparison group, with TDC, ID, ASD, ADHD and Turner syndrome all included in studies in this review. This makes an accurate comparison between the studies more difficult. For example, a difference between the FXS and Turner syndrome groups provides insight into how autonomic function can differ between genetic‐based conditions, whereas a between‐groups difference between the FXS and TDC groups provides more insight into how autonomic function differs in FXS from the general population. These are important considerations for interpreting the results of the studies included in this review. It is recommended that a TDC group be utilised alongside other comparison groups, that is, ID, Turner syndrome, in the future, to ensure accurate comparison can be made in systematic reviews and meta‐analyses.

The clinical relevance of autonomic dysfunction in FXS can be explained by the neurovisceral integration model (Thayer and Lane 2000). This model describes a functional and structural network that links autonomic function with attentional and affective systems, demonstrating associations with underactivity of the parasympathetic nervous system or overactivity of the sympathetic nervous system, which can both be attributed to hyperarousal, with anxiety and emotional dysregulation (Chevalère et al. 2019; Thayer and Lane 2009). As discussed earlier in this review, anxiety is commonly observed in FXS (Cordeiro et al. 2011). The frequent finding of autonomic hyperarousal in FXS from this systematic review, alongside existing literature describing an anxiety phenotype in FXS, supports the link between physiological arousal and affective regulation proposed by the neurovisceral integration model. This suggests that autonomic dysfunction, in the presence of underactivity of the parasympathetic nervous system and/or overactivity of the sympathetic nervous system, may be associated with anxiety in FXS. Further links with neurophysiological pathways may explain the mechanism behind autonomic hyperarousal in FXS. For example, brain regions (i.e., amygdala and medial prefrontal cortex) suspected to be disrupted in FXS are involved in both emotion and fear processing as well as autonomic regulation (Kramvis et al. 2020; Olmos‐Serrano and Corbin 2011). It is possible that altered processing within such pathways may contribute to autonomic hyperarousal observed in FXS.

Determining the autonomic profile in FXS can be clinically useful, particularly in future investigations of therapeutic interventions. Measures of autonomic function could serve as objective biomarkers in clinical trials of pharmacological and/or behavioural interventions for FXS. Autonomic dysfunction may also point towards potential therapeutic interventions for those with FXS. Interestingly, preliminary success in reducing challenging behaviours in another genetic‐based ID (Prader–Willi syndrome) was achieved through transcutaneous vagal nerve stimulation (tVNS). A review highlights empirical findings from tVNS research in PWS, demonstrating that challenging behaviours improved alongside improvements in overall global functioning in PWS after a treatment course of tVNS (Holland and Manning 2022).

### 4.1 Quality Discussion

Although most studies accounted for age and gender, either via proactive matching of groups or retroactive statistical accounting for confounding variables, few studies described or accounted for medication use. We acknowledge that it is difficult to recruit accordingly or to statistically account for medication use, given the variation in the number and types of medications prescribed for individuals with FXS. It is important for researchers to consider the frequently used medications by individuals with FXS, such as stimulants and alpha‐2 agonists, which affect autonomic function. For example, HRV is significantly reduced in individuals using psychotropic medications (Alvares et al. 2016). We further recommend that authors employ strategies to deal with age and gender, given that the clinical presentation of FXS can be affected by both variables (Gabis et al. 2011).

### 4.2 Study Limitations and Future Directions

Reviewing existing literature examining autonomic function in FXS was complex, given the varying comparison groups recruited, age ranges of participants and physiological recording techniques/methodology used. Therefore, a meta‐analysis could not be conducted across all these data. The conclusions drawn from this review are limited by the clinical heterogeneity within FXS. Specifically, the wide spectrum of cognitive and behavioural impairments, particularly within males with FXS, indicates the need for sensitive study designs and analysis accounting for such variation. Furthermore, the variation in study designs, including case control studies, cohort studies, longitudinal studies and RCTs, hinders our ability to compare the quality between all identified studies. It would be prudent to consider the study design of potential articles as an inclusion and/or exclusion criteria in future systematic reviews and meta‐analyses of autonomic function in FXS. Future research is needed examining autonomic function in FXS with defined age groups, including examinations of exclusively adult populations. There were few longitudinal studies examining how autonomic function may change with age in FXS. Further investigations of autonomic function in additional genetic‐based IDs, such as Williams syndrome, Prader–Willi syndrome and *SYNGAP1*‐related ID, will shed light on whether autonomic function is a common phenotype across IDs which have a genetic aetiology. If the research continues to support the neurovisceral integration model, it is further possible that autonomic function may serve as a therapeutic target for interventions aimed to reduce the burden of cognitive and affective symptoms in FXS and genetic‐based IDs.

## 5 Conclusion

Our findings suggest that autonomic dysfunction, and more specifically autonomic hyperarousal, is a part of the clinical phenotype of FXS. Notably, the findings indicate that there may be gender‐specific differences in autonomic function in FXS. Overall, the results provide insight for potential biomarkers for clinicians and those researching therapeutic targets for FXS and provide an explanation, in part, for affective dysregulation in FXS.

## Funding

This article was funded by the Winefride and Booth Smith Studentship at the University of Edinburgh.

## Conflicts of Interest

The authors declare no conflicts of interest.

## Supporting information




**Data S1:** Supporting Information.

## Data Availability

The data that support the findings of this study are available from the corresponding author upon reasonable request.
