# Supplementary material for: Autonomic Function in Fragile X Syndrome: A Systematic Review
Source: J Intellect Disabil Res. 2026 Apr 24;70(8):773–87. doi: 10.1111/jir.70106 (PMC13327213; doi:10.1111/jir.70106)
Supplement: Supplementary file 1 — Data S1: Supporting Information. [file JIR-70-773-s001.docx]

**Supplementary Material**

1. **Search**

**EMBASE Search:**

("Autonomic nervous system" or "autonomic regulation" or "autonomic activity" or "autonomic function" or "sympathetic nervous system" or "sympathetic regulation" or "sympathetic activation" or "parasympathetic nervous system" or "parasympathetic regulation" or "parasympathetic activation" or "vagal nerve" or "vagal nerve activity" or "vagal parasympathetic" or "vagal tone" or "respiratory sinus arrythmia" or "pre-ejection period" or "preejection period" or "heart rate var*" or "HRV" or "skin conductance" or "psychogalvanic response" or "psycho-galvanic response" or "psychogalvanic reflex" or "psycho-galvanic reflex" or "electro-dermal activity" or "electrodermal" or "electro-dermal response" or "pupil dilation" or "pupillometry").mp. [mp=title, book title, abstract, original title, name of substance word, subject heading word, floating sub-heading word, keyword heading word, organism supplementary concept word, protocol supplementary concept word, rare disease supplementary concept word, unique identifier, synonyms, population supplementary concept word, anatomy supplementary concept word]

autonomic nervous system/

human/ or fragile X syndrome/ or fragile X mental retardation protein/

intellectual impairment/

("Fragile X Syndrome" or "Fragile X" or "FXS" or "FMR1" or "FMR-1" or "intellectual disabilit*" or "ID" or "learning differences" or "learning disabilities" or "mental retardation").mp. [mp=title, book title, abstract, original title, name of substance word, subject heading word, floating sub-heading word, keyword heading word, organism supplementary concept word, protocol supplementary concept word, rare disease supplementary concept word, unique identifier, synonyms, population supplementary concept word, anatomy supplementary concept word]

limit 5 to yr="1991 -Current"

1 or 2

3 or 4 or 5

6 and 7 and 8

**MEDLINE Search:**

Autonomic Nervous System/

Humans/ or Fragile X Syndrome/ or Fragile X Mental Retardation Protein/

Intellectual Disability/

("Autonomic nervous system" or "autonomic regulation" or "autonomic activity" or "autonomic function" or "sympathetic nervous system" or "sympathetic regulation" or "sympathetic activation" or "parasympathetic nervous system" or "parasympathetic regulation" or "parasympathetic activation" or "vagal nerve" or "vagal nerve activity" or "vagal parasympathetic" or "vagal tone" or "respiratory sinus arrythmia" or "pre-ejection period" or "preejection period" or "heart rate var*" or "HRV" or "skin conductance" or "psychogalvanic response" or "psycho-galvanic response" or "psychogalvanic reflex" or "psycho-galvanic reflex" or "electro-dermal activity" or "electrodermal" or "electro-dermal response" or "pupil dilation" or "pupillometry").mp. [mp=title, book title, abstract, original title, name of substance word, subject heading word, floating sub-heading word, keyword heading word, organism supplementary concept word, protocol supplementary concept word, rare disease supplementary concept word, unique identifier, synonyms, population supplementary concept word, anatomy supplementary concept word]

("Fragile X Syndrome" or "Fragile X" or "FXS" or "FMR1" or "FMR-1" or "intellectual disabilit*" or "ID" or "learning differences" or "learning disabilities" or "mental retardation").mp. [mp=title, book title, abstract, original title, name of substance word, subject heading word, floating sub-heading word, keyword heading word, organism supplementary concept word, protocol supplementary concept word, rare disease supplementary concept word, unique identifier, synonyms, population supplementary concept word, anatomy supplementary concept word]

limit 5 to yr="1991 -Current"

1 or 4

2 or 3 or 5

6 and 7 and 8

**PsychINFO Search:**

exp Heart Rate/ or exp Autonomic Nervous System/ or exp Heart Rate Variability/

exp Fragile X Syndrome/ or exp Intellectual Development Disorder/

("Autonomic nervous system" or "autonomic regulation" or "autonomic activity" or "autonomic function" or "sympathetic nervous system" or "sympathetic regulation" or "sympathetic activation" or "parasympathetic nervous system" or "parasympathetic regulation" or "parasympathetic activation" or "vagal nerve" or "vagal nerve activity" or "vagal parasympathetic" or "vagal tone" or "respiratory sinus arrythmia" or "pre-ejection period" or "preejection period" or "heart rate var*" or "HRV" or "skin conductance" or "psychogalvanic response" or "psycho-galvanic response" or "psychogalvanic reflex" or "psycho-galvanic reflex" or "electro-dermal activity" or "electrodermal" or "electro-dermal response" or "pupil dilation" or "pupillometry").mp. [mp=title, book title, abstract, original title, name of substance word, subject heading word, floating sub-heading word, keyword heading word, organism supplementary concept word, protocol supplementary concept word, rare disease supplementary concept word, unique identifier, synonyms, population supplementary concept word, anatomy supplementary concept word]

("Fragile X Syndrome" or "Fragile X" or "FXS" or "FMR1" or "FMR-1" or "intellectual disabilit*" or "ID" or "learning differences" or "learning disabilities" or "mental retardation").mp. [mp=title, book title, abstract, original title, name of substance word, subject heading word, floating sub-heading word, keyword heading word, organism supplementary concept word, protocol supplementary concept word, rare disease supplementary concept word, unique identifier, synonyms, population supplementary concept word, anatomy supplementary concept word]

limit 4 to yr="1991 -Current"

1 or 3

2 or 4

5 and 6 and 7

**Web of Science Search:**

**(TS=(“Autonomic nervous system” OR “autonomic regulation” OR “autonomic activity” OR “autonomic function” OR “sympathetic nervous system” OR “sympathetic regulation” OR “sympathetic activation” OR “parasympathetic nervous system” OR “parasympathetic regulation” OR “parasympathetic activation” OR “vagal nerve” OR “vagal nerve activity” OR “vagal parasympathetic” OR “vagal tone” OR “respiratory sinus arrythmia” OR “pre-ejection period” OR “preejection period” OR “heart rate var*” OR “HRV” OR “skin conductance” OR “psychogalvanic response” OR “psycho-galvanic response” OR “psychogalvanic reflex” OR “psycho-galvanic reflex” OR “electro-dermal activity” OR “electrodermal” OR “electro-dermal response” OR “pupil dilation” OR “pupillometry”))**

**TS=(“Fragile X Syndrome” OR “Fragile X” OR “FXS” OR “FMR1” OR “FMR-1” OR “intellectual disabilit*” OR “ID” OR “learning differences” OR “learning disabilities” OR “mental retardation”)**

**#1 AND #2**

1. **Quality Assessment Table**

|  | **Is the case definition adequate? / ascertainment of exposure** | **Representativeness of the cases** | **Selection & Definition of Controls** | **Comparability of cases and controls ^a^** | **Same method of ascertainment for cases and controls** | **Non-Response rate** |
| --- | --- | --- | --- | --- | --- | --- |
| (Belser & Sudhalter, 1995) | no description | potential for selection biases or not stated | controls with a different condition* | Gender | Yes* | rate different and no designation |
| (Black et al., 2021) | yes, with independent validation (i.e. genetic test)* | consecutive or obviously representative series of cases* | typically developing controls* | Age, gender | Yes* | same rate for both groups* |
| (Boccia & Roberts, 2000) | no description | consecutive or obviously representative series of cases* | typically developing controls* | Age, gender | Yes* | same rate for both groups* |
| (Cohen et al., 2015) | yes, with independent validation (i.e. genetic test)* | consecutive or obviously representative series of cases* | typically developing controls & controls with a different condition* | Age, gender, medication* | Yes* | non respondents described |
| (Ezell et al., 2021) | Yes; based on self/caregiver reports | consecutive or obviously representative series of cases* | typically developing controls & controls with a different condition* | Age | Yes* | same rate for both groups* |
| (Farzin et al., 2009) | yes, with independent validation (i.e. genetic test)* | consecutive or obviously representative series of cases* | typically developing controls* | Age, gender, medication* | Yes* | same rate for both groups* |
| (Farzin et al., 2011) | yes, with independent validation (i.e. genetic test)* | consecutive or obviously representative series of cases* | typically developing controls* | Medication | Yes* | same rate for both groups* |
| (Hagerman et al., 2002) | yes, with independent validation (i.e. genetic test)* | consecutive or obviously representative series of cases* | typically developing controls* | Age, medication | Yes* | same rate for both groups* |
| (Hall et al., 2009) | yes, with independent validation (i.e. genetic test)* | consecutive or obviously representative series of cases* | typically developing controls* | Age, gender, medication* | Yes* | same rate for both groups* |
| (Heilman et al., 2011) | yes, with independent validation (i.e. genetic test)* | consecutive or obviously representative series of cases* | typically developing controls* | Age, gender, medication* | Yes* | non respondents described |
| (Hogan et al., 2021) | yes, with independent validation (i.e. genetic test)* | consecutive or obviously representative series of cases* | typically developing controls* | Age, gender | Yes* | rate different and no designation |
| (Keysor et al., 2002) | yes, with independent validation (i.e. genetic test)* | consecutive or obviously representative series of cases* | typically developing controls & controls with a different condition* | Age, gender, medication* | Yes* | same rate for both groups* |
| (Klusek et al., 2013) | no description | potential for selection biases or not stated | typically developing controls & controls with a different condition* | Age, gender, medication* | Yes* | same rate for both groups* |
| (Li et al., 2022) | no description | consecutive or obviously representative series of cases* | typically developing controls* | Age, gender | Yes* | rate different and no designation |
| (L.J. Miller et al., 1999) | yes, with independent validation (i.e. genetic test)* | consecutive or obviously representative series of cases* | typically developing controls* | Age, gender | No | same rate for both groups* |
| (Miller et al., 2023) | yes, with independent validation (i.e. genetic test)* | consecutive or obviously representative series of cases* | controls with a different condition* | Age, gender, medication* | Yes* | non respondents described |
| (Roberts et al., 2001) | no description | consecutive or obviously representative series of cases* | typically developing controls* | Age, gender, medication* | Yes* | rate different and no designation |
| (Roberts et al., 2006) | no description | consecutive or obviously representative series of cases* | typically developing controls* | Age, gender | Yes* | rate different and no designation |
| (Roberts et al., 2008) | no description | consecutive or obviously representative series of cases* | typically developing controls & controls with a different condition* | Age, gender | Yes* | same rate for both groups* |
| (Roberts, Hatton, et al., 2012) | yes, with independent validation (i.e. genetic test)* | consecutive or obviously representative series of cases* | typically developing controls* | Age, gender | Yes* | same rate for both groups* |
| (Roberts, Tonnsen, et al., 2012) | yes, with independent validation (i.e. genetic test)* | potential for selection biases or not stated | typically developing controls* | Age, gender | Yes* | same rate for both groups* |
| (Roberts et al., 2013) | yes, with independent validation (i.e. genetic test)* | consecutive or obviously representative series of cases* | typically developing controls* | Age, gender, medication* | Yes* | same rate for both groups* |
| (Tonnsen et al., 2013) | yes, with independent validation (i.e. genetic test)* | consecutive or obviously representative series of cases* | typically developing controls* | Age, gender | Yes* | non respondents described |
| (Wall & Roberts, 2023) | yes, with independent validation (i.e. genetic test)* | consecutive or obviously representative series of cases* | typically developing controls & controls with a different condition* | Age, gender | Yes* | same rate for both groups* |
| (Williams et al., 2013) | yes, with independent validation (i.e. genetic test)* | consecutive or obviously representative series of cases* | typically developing controls* | Age, gender | Yes* | same rate for both groups* |

Note: only case control studies were assessed.

1. Confounders to be matched for or controlled (deemed important for this type of study): age, gender & medication usage; star only given if all 3 are reported.
2. **Prisma Checklist**

| **Section and Topic** | **Item #** | **Checklist item** | **Location where item is reported** |
| --- | --- | --- | --- |
| **TITLE** | | |  |
| Title | 1 | Identify the report as a systematic review. | Title |
| **ABSTRACT** | | |  |
| Abstract | 2 | See the PRISMA 2020 for Abstracts checklist. | Abstract |
| **INTRODUCTION** | | |  |
| Rationale | 3 | Describe the rationale for the review in the context of existing knowledge. | Introduction (p1-2) |
| Objectives | 4 | Provide an explicit statement of the objective(s) or question(s) the review addresses. | Introduction (p2) |
| **METHODS** | | |  |
| Eligibility criteria | 5 | Specify the inclusion and exclusion criteria for the review and how studies were grouped for the syntheses. | Methods (p2) |
| Information sources | 6 | Specify all databases, registers, websites, organisations, reference lists and other sources searched or consulted to identify studies. Specify the date when each source was last searched or consulted. | Methods (p2) |
| Search strategy | 7 | Present the full search strategies for all databases, registers and websites, including any filters and limits used. | Methods (p2); supplementary material |
| Selection process | 8 | Specify the methods used to decide whether a study met the inclusion criteria of the review, including how many reviewers screened each record and each report retrieved, whether they worked independently, and if applicable, details of automation tools used in the process. | Methods (p2-3) |
| Data collection process | 9 | Specify the methods used to collect data from reports, including how many reviewers collected data from each report, whether they worked independently, any processes for obtaining or confirming data from study investigators, and if applicable, details of automation tools used in the process. | Methods (p2-3) |
| Data items | 10a | List and define all outcomes for which data were sought. Specify whether all results that were compatible with each outcome domain in each study were sought (e.g. for all measures, time points, analyses), and if not, the methods used to decide which results to collect. | Methods (p3) |
|  | 10b | List and define all other variables for which data were sought (e.g. participant and intervention characteristics, funding sources). Describe any assumptions made about any missing or unclear information. | Methods (p3) |
| Study risk of bias assessment | 11 | Specify the methods used to assess risk of bias in the included studies, including details of the tool(s) used, how many reviewers assessed each study and whether they worked independently, and if applicable, details of automation tools used in the process. | Methods (p3) |
| Effect measures | 12 | Specify for each outcome the effect measure(s) (e.g. risk ratio, mean difference) used in the synthesis or presentation of results. | N/A |
| Synthesis methods | 13a | Describe the processes used to decide which studies were eligible for each synthesis (e.g. tabulating the study intervention characteristics and comparing against the planned groups for each synthesis (item #5)). | Methods (p3); supplementary material |
|  | 13b | Describe any methods required to prepare the data for presentation or synthesis, such as handling of missing summary statistics, or data conversions. | N/A |
|  | 13c | Describe any methods used to tabulate or visually display results of individual studies and syntheses. | Methods (p3) |
|  | 13d | Describe any methods used to synthesize results and provide a rationale for the choice(s). If meta-analysis was performed, describe the model(s), method(s) to identify the presence and extent of statistical heterogeneity, and software package(s) used. | Methods (p2-3) |
|  | 13e | Describe any methods used to explore possible causes of heterogeneity among study results (e.g. subgroup analysis, meta-regression). | N/A |
|  | 13f | Describe any sensitivity analyses conducted to assess robustness of the synthesized results. | N/A |
| Reporting bias assessment | 14 | Describe any methods used to assess risk of bias due to missing results in a synthesis (arising from reporting biases). | N/A |
| Certainty assessment | 15 | Describe any methods used to assess certainty (or confidence) in the body of evidence for an outcome. | N/A |
| **RESULTS** | | |  |
| Study selection | 16a | Describe the results of the search and selection process, from the number of records identified in the search to the number of studies included in the review, ideally using a flow diagram. | Results (p3) |
|  | 16b | Cite studies that might appear to meet the inclusion criteria, but which were excluded, and explain why they were excluded. | N/A |
| Study characteristics | 17 | Cite each included study and present its characteristics. | Results: Tables 1 & 2 (p5-7; p10-12) |
| Risk of bias in studies | 18 | Present assessments of risk of bias for each included study. | Results (p13); supplementary material |
| Results of individual studies | 19 | For all outcomes, present, for each study: (a) summary statistics for each group (where appropriate) and (b) an effect estimate and its precision (e.g. confidence/credible interval), ideally using structured tables or plots. | Results (p3-13) |
| Results of syntheses | 20a | For each synthesis, briefly summarise the characteristics and risk of bias among contributing studies. | Results (p3-13) |
|  | 20b | Present results of all statistical syntheses conducted. If meta-analysis was done, present for each the summary estimate and its precision (e.g. confidence/credible interval) and measures of statistical heterogeneity. If comparing groups, describe the direction of the effect. | Results (p3-13) |
|  | 20c | Present results of all investigations of possible causes of heterogeneity among study results. | Results (p3-13) |
|  | 20d | Present results of all sensitivity analyses conducted to assess the robustness of the synthesized results. | N/A |
| Reporting biases | 21 | Present assessments of risk of bias due to missing results (arising from reporting biases) for each synthesis assessed. | N/A |
| Certainty of evidence | 22 | Present assessments of certainty (or confidence) in the body of evidence for each outcome assessed. | N/A |
| **DISCUSSION** | | |  |
| Discussion | 23a | Provide a general interpretation of the results in the context of other evidence. | Discussion (p13-16) |
|  | 23b | Discuss any limitations of the evidence included in the review. | Discussion (p16) |
|  | 23c | Discuss any limitations of the review processes used. | Discussion (p16) |
|  | 23d | Discuss implications of the results for practice, policy, and future research. | Discussion (p13-16) |
| **OTHER INFORMATION** | | |  |
| Registration and protocol | 24a | Provide registration information for the review, including register name and registration number, or state that the review was not registered. | Methods (p2) |
|  | 24b | Indicate where the review protocol can be accessed, or state that a protocol was not prepared. | Methods (p2) |
|  | 24c | Describe and explain any amendments to information provided at registration or in the protocol. | Methods (p2) |
| Support | 25 | Describe sources of financial or non-financial support for the review, and the role of the funders or sponsors in the review. | Discussion (p16) |
| Competing interests | 26 | Declare any competing interests of review authors. | Title Page |
| Availability of data, code and other materials | 27 | Report which of the following are publicly available and where they can be found: template data collection forms; data extracted from included studies; data used for all analyses; analytic code; any other materials used in the review. | Methods, supplementary material |

*From:*  Page MJ, McKenzie JE, Bossuyt PM, Boutron I, Hoffmann TC, Mulrow CD, et al. The PRISMA 2020 statement: an updated guideline for reporting systematic reviews. BMJ 2021;372:n71. doi: 10.1136/bmj.n71. This work is licensed under CC BY 4.0. To view a copy of this license, visit <https://creativecommons.org/licenses/by/4.0/>
